# A “Mix and Match” in Hemochromatosis—A Case Report and Literature Focus on the Liver

**DOI:** 10.3390/medicina59091586

**Published:** 2023-09-01

**Authors:** Claudia Oana Cobilinschi, Ioana Săulescu, Simona Caraiola, Andra Florina Nițu, Radu Lucian Dumitru, Ioana Husar-Sburlan, Andra Rodica Bălănescu, Daniela Opriș-Belinski

**Affiliations:** 1 Department of Rheumatology and Internal Medicine, Carol Davila University of Medicine and Pharmacy, 050474 Bucharest, Romania; claudia.cobilinschi@umfcd.ro (C.O.C.); ioana_oprisan@yahoo.com (I.S.); scaraiola@yahoo.com (S.C.); balanescu.andra@gmail.com (A.R.B.); danaopris0103@yahoo.com (D.O.-B.); 2 Department of Rheumatology and Internal Medicine, Sfânta Maria Clinical Hospital Bucharest, 011172 Bucharest, Romania; 3 Department of Internal Medicine, Colentina Clinical Hospital, Bucharest, 020125 Bucharest, Romania; 4 Department of Radiology and Medical Imaging, Sf Maria Clinical Hospital Bucharest, 011172 Bucharest, Romania; andra_florina_nitu@yahoo.com; 5 Department of Gastroenterology, Sf Maria Clinical Hospital Bucharest, 011172 Bucharest, Romania; sburlan.ioana@gmail.com

**Keywords:** hemochromatosis, cirrhosis, excess iron, severe arthritis, phlebotomy

## Abstract

Hemochromatosis is a genetic disorder characterized by increased iron storage in various organs with progressive multisystemic damage. Despite the reports dating back to 1865, the diagnosis of hemochromatosis poses a challenge to clinicians due to its non-specific symptoms and indolent course causing significant delay in disease recognition. The key organ that is affected by iron overload is the liver, suffering from fibrosis, cirrhosis or hepatocellular carcinoma, complications that can be prevented via early diagnosis and treatment. This review aims to draw attention to the pitfalls in diagnosing hemochromatosis. We present a case with multiorgan complaints, abnormal iron markers and a consistent genetic result. We then examine the relevant literature and discuss hemochromatosis subtypes and liver involvement, including transplant outcome and treatment options. In summary, hemochromatosis remains difficult to diagnose due to its symptom heterogeneity and rarity; thus, further education for practitioners of all disciplines is useful in facilitating its early recognition and management.

## 1. Introduction

Hemochromatosis describes a condition characterized by iron overload which leads to organ deposition and impairment consequent to cellular destruction. Amongst them, the liver, joints, heart, and glands such as the thyroid or pituitary gland predominantly suffer from excess iron [1]. Patients can display a variety of clinical features, depending on the amount of iron overload. However, the most common symptoms are chronic fatigue, a bronzed skin appearance, joint pain, swelling, or stiffness. Organ toxicity translates into liver dysfunction (hepatomegaly, cirrhosis, and carcinoma), diabetes, cardiac involvement (cardiomyopathy and arrhythmias), or hypogonadism [2].

Patients with iron overload can exhibit an array of symptoms, depending on the targeted organ, as summarized below [3]:

- Liver: abnormal liver enzymes, fibrosis, cirrhosis, and hepatocellular carcinoma;
- Glands: diabetes, hypothyroidism, and hypopituitarism;
- Musculoskeletal: osteopenia/osteoporosis, arthralgias, osteoarthritis, and calcium pyrophosphate-induced arthropathy;
- Nervous system: fatigue, and lethargy;
- Skin: hyperpigmentation;
- Reproduction: hypogonadism, low libido, impotence, and dis/amenorrhea;
- Heart: congestive heart failure, cardiomyopathy, and arrythmias.

Hemochromatosis can be either hereditary (HH) or acquired, and is acquired secondary to diseases that disturb the iron metabolism. The latter can be due to thalassemia, sickle cell disease, pyruvate kinase deficiency, supplement or alcohol consumption, and conditions that require repeated blood transfusions. Hereditary hemochromatosis is transmitted in an autosomal-recessive manner, and this is due to a mutation of the hemochromatosis gene named HFE, located on the sixth chromosome. The most frequent abnormality is displayed on H63D (the substitution of histidine with aspartic acid at position 63) or C282Y (the replacement of cysteine by tyrosine at position 282) of the HFE gene, but rarer variants exist, classifying HH into distinct subgroups [4]. Type 1 hemochromatosis is caused by a HFE mutation and patients most commonly experience a homozygosity of C282Y (83%) or compound heterozygosity of C282/H63D. This subtype affects adults who can present disease-related symptoms when aged 40–60 or after menopause in females. Type 2 hemochromatosis can occur due to mutations of the HJV (hemojuvenil BMP Co-receptor) or HAMP (hepicidin antimicrobial peptide) genes. It is also called the juvenile form since it can have a childhood onset and by young adulthood the secretion of sex hormones is insufficient due to iron excess, causing puberty-related issues and premature amenorrhea. In the third type of hemochromatosis (type 3), mutation in the TFR2 (transferrin receptor 2) gene was detected and symptoms can begin after the first two decades. Type 4 hemochromatosis or the ferroportin disease has a mutated gene, SLC40A1 (solute carrier family 40 member 1), which leads to hepcidin binding impairment due to ferroportin [5]. Depending on the gene involved, clinical features differ indicating the significance of the genotype–phenotype link.

The epidemiology of the genetic disorder confirms that males are more affected than are females, with an estimated 1.8–3 to 1 ratio, and that they are more prone to exhibiting more severe liver disease. Despite initially being described as rare, HH appears to have a prevalence of up to 0.7 within the European population, while the HFE gene mutation is identified in around 5% of patients of European origin [6].

HH can be responsible for severe liver disease that can rapidly progress to cirrhosis or hepatocellular carcinoma (HCC) with potentially fatal outcomes. Diagnosing HH poses a challenge in clinical practice for various reasons. Genetic testing is not widely available and rarer mutations can be often overlooked [7]. Moreover, patients display a heterogeneity of symptoms depending on organ involvement that can easily mislead the diagnosis. HH can remain asymptomatic for a considerable period. When the clinical setting becomes evocative, patients are usually in an advanced disease state with significantly high iron deposits. Imaging brings additional value to diagnosis since magnetic resonance imaging (MRI) has become a gold-standard method to detect and quantify iron overload in different organs [8]. Organ biopsy remains a useful tool in selected patients in which additional liver conditions are suspected if ferritin levels are persistently higher than 1000 ng/mL or if genetic testing results are negative despite iron overload marker abnormalities [9].

Thus, diagnosing HH in a presymptomatic state can be the key to successful patient management and limiting severe organ damage. Thus, novel biomarkers could help to improve the diagnostic strategy [10,11]. This paper aims to raise awareness of HH and the available tools for diagnosis and to consider this genetic disorder in the presence of unspecific, multisystemic complaints by bringing forward the latest data in the field.

## 2. Case Presentation

A 56-year-old male patient was admitted in the Rheumatology Department of a second-care facility for diffuse abdominal pain, increasing fatigue, and painful swelling of the second and third metacarpophalangeal joints in both hands. He reported the onset of joint symptoms, including bilateral knee and ankle two years back when a diagnosis of gout was made, and specific therapy was initiated without significant benefits.

Apart from a history of stage III arterial hypertension, which was well controlled by chronic medication, the patient was newly diagnosed with type 2 diabetes for which he was prescribed metformin daily. No other drugs were used. He denies smoking or being exposed to toxins but confirms occasional alcohol drinking. Family history was not suggestive of chronic diseases.

**Figure 1:**
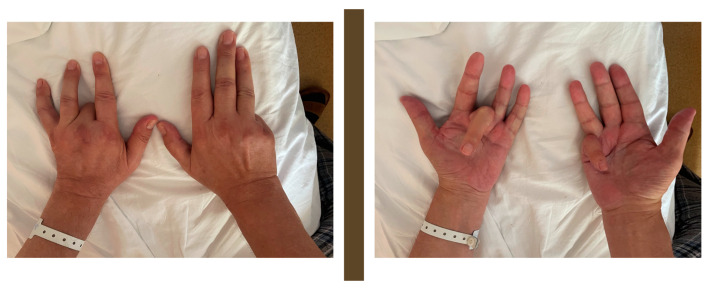
Bilateral Dupuytren’s contracture and skin hyperpigmentation.

A clinical examination of the patient revealed a severe bilateral deforming Dupuytren’s contracture (Figure 1), symmetric gynecomastia, and apparent hyperpigmentation of sun-exposed skin (face and hands). Moreover, a mildly enlarged spleen was noted, as was pain upon palpation in the right hypochondriac area. Later, the patient confessed to having a low libido for the last year.

**Figure 2:**
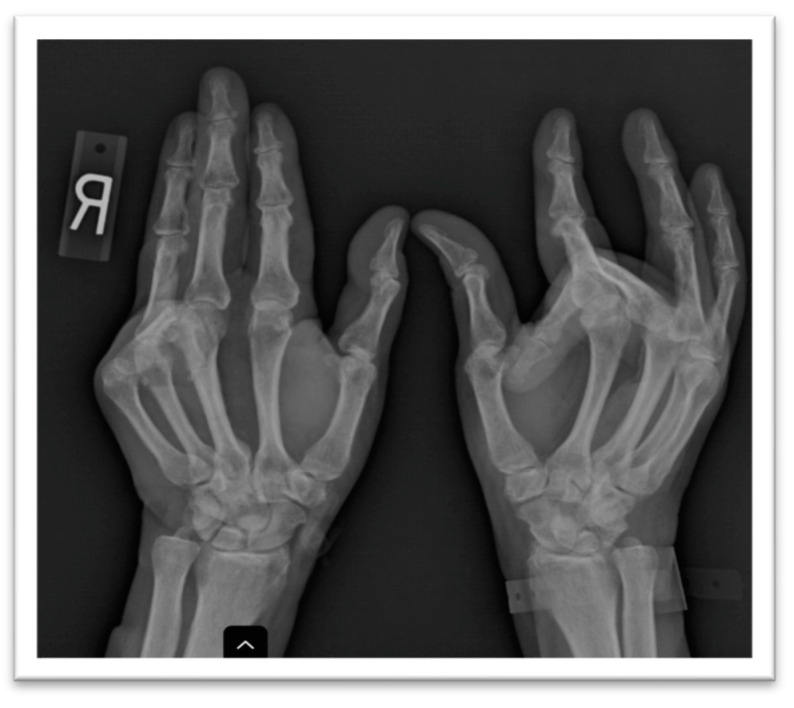
Plain X-ray of hands showing prominent interphalangeal bony productions and subluxations of multiple digits.

A plain X-ray of the hands showed juxta articular osteopenia, the large bony proliferation of the proximal interphalangeal joints, the erosion of this site in the fourth digit of the left hand, and the fixed flexion of two digits with subluxation (Figure 2). A hand ultrasound confirmed the presence of osteophytes defined by a step-up type of lesion, one large erosion in the right hand and signs of calcium pyrophosphate dehydrate deposition. A foot ultrasound indicated the presence of the double-contour sign at the first metatarsal, compatible with the patient’s previous diagnosis of gout. The hand joint lesions were indicative of destructive osteoarthritis (OA) but with atypical distribution; thus, more tests were deemed necessary to confirm the diagnosis and etiology.

Several differential diagnoses were taken into account considering the patient’s presentation upon admission, such as rheumatoid arthritis, since the patient suffered from a painful polyarticular pattern and a limited range of motion in his hands. A bilateral Dupuytren’s contracture can occur due to alcohol consumption, diabetes or vitamin D deficiency and these could have partly explained the patient’s asthenia. We also thought of viral hepatitis due to abdominal pain, joint involvement and hyperpigmentation of the skin.

During his admission, he displayed no increase in inflammatory markers, negative rheumatoid factor and anti-citrullinated protein antibodies (ACPA), slight hypovitaminosis D and mild thrombocytopenia but significantly increased cholestasis, reaching three times the reference value (alkaline phosphatase PAL, gamma glutamyl transferase GGT and total bilirubin levels). The lipid profile (total cholesterol and fractions) was within the normal range, and so was the reticulocyte count and blood sugar.

**Figure 3:**
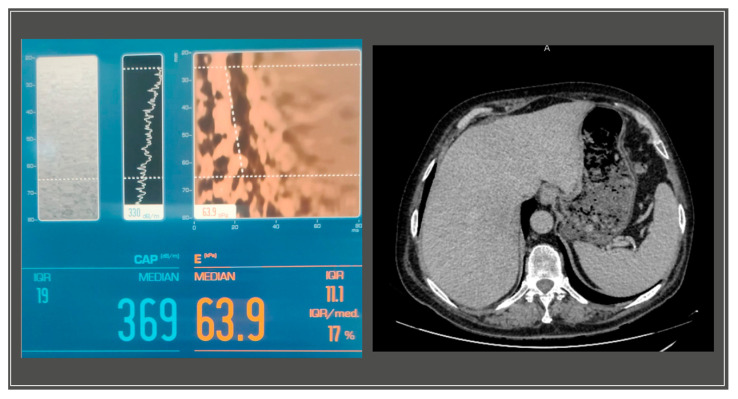
Liver imaging with S3F4 score on Fibroscan® and abdominal CT with mild liver enlargement.

A gastroenterology evaluation was requested at the time; the abdominal ultrasound revealed a liver with a hyperechoic structure, with considerable steatosis, a nodular structure, with slightly irregular edges, an abnormal vascular system with reduced blood supply and a disrupted flow pattern in the periphery, without intrahepatic localized processes. The pancreas showed no anomalies, while the spleen was slightly increased in size, measuring 14 cm in its long axis. The further Fibroscan^®^ test displayed stage III steatosis and grade IV liver fibrosis (S3, F4) (Figure 3).

An upper endoscopy identified mild gastritis and the presence of a Schatzi ring.

We completed blood tests in order to investigate the liver dysfunction, testing for antinuclear antibodies, antimitochondrial (AMA) and anti-smooth muscle antibodies (ASMA), and anti-liver-kidney microsomal antibodies (LKM)-1 but they all came back negative, and so did the screening for viral hepatitis B, C, and alpha-fetoprotein. Contrast computed tomography showed a relatively homogeneous liver, except for some microcalcifications and a larger spleen of 13.7 cm.

Since the patient’s joint complaints were persistent despite usual analgesia, a plain X-ray was performed showing large bony productions of the second and fourth left proximal interphalangeal joint, an erosion of the right metacarpal bone, multiple subluxations, subchondral sclerosis and the fixed flexion of two of the digits, corresponding to a clinical bilateral Dupuytren’s contracture.

The iron level was tested with the result of 212 μg/dL (normal reference range 40–120 μg/dL), the ferritin value was 539 ng/mL (the normal laboratory value is between 25 and 350 ng/mL) and the transferrin saturation coefficient was 73.56% (normal range 16–45%). No folate or B12 vitamin deficiency was identified. Sickle cell disease and beta-thalassemia were excluded through hemoglobin electrophoresis and a blood smear. Thyroid function showed a mild degree of hypothyroidism with negative antithyroid autoantibodies. Given the abnormal values of iron markers, genetic testing was performed for the suspicion of HH. Mutations of C282Y and H63D on the HFE gene were analyzed through the restriction fragment length polymorphism (RFLP) method and the patient displayed C282Y/H63D compound heterozygosity. No other rarer variants were available for testing.

Since no available diagnostic or classification criteria can be used in HH, the most recent algorithm issued by the European Association for the Study of the Liver (EASL) 2022 was used. Thus, clinical symptoms (fatigue, arthralgia, and arthritis, liver disease, and diabetes), and an iron panel (a high transferrin saturation and serum ferritin) led to further HFE genetic testing. Since the patient was C282Y/H63D-heterozygous, an abdominal MRI was deemed necessary for iron deposition identification. The MRI was positive for diffuse liver siderosis as determined via a pattern of global decrease in signal in the hepatic parenchyma in the “in-phase” gradient as opposed to the “out-of-phase” sequence, which highly corresponds to a HH diagnosis. Quantifying the iron concentration was not possible due to an unspecific MRI protocol. A liver biopsy was not performed due to the lack of patient compliance.

Following the diagnosis, the patient underwent cardiac evaluation showing no signs of dilated cardiomyopathy, heart failure, or conduction anomalies.

The patient was referred to the hematologist for weekly phlebotomy sessions per the induction phase with a marked improvement in clinical symptoms, including arthralgias and fatigue, and in biological parameters with a normalization of iron excess markers, as seen in Table 1.

## 3. Discussion and Literature Overview

Hemochromatosis is a metabolic storage disease characterized by iron overload targeting the main organs such as the liver, pancreas, joints, heart, nervous system, thyroid, and parathyroid glands. As such, middle-aged patients can present with diabetes, arthropathy, cardiac failure, hypogonadism, or hypothyroidism. The most severe injury is related to the liver where deposition can cause liver fibrosis, cirrhosis, or even hepatocellular carcinoma in later stages [12]. Iron metabolism is tightly controlled in the body by the hepcidinferroportin axis since its presence is essential on both cellular and systemic levels. Given that hemoglobin formation relies on iron, its role is undeniable in essential physiological processes such as respiration, and metabolic and defense reactions. The digestive system takes up to 1–2 mg of iron daily but most of it resides in erythrocytes that eventually become phagocyted by macrophages. Hepcidine is the key component that controls iron homeostasis through its action on gut absorption, plasma levels and tissue storage with the aid of ferroportin [13]. Excess iron is caused by higher digestive metal absorption and increased iron discharge from macrophages, leading to significantly high iron levels as reflected via transferrin saturation. Hepcidin, which is the main iron-negative regulator in conjunction with the receptor ferroportin, is able to limit iron intake in the duodenum and its release from macrophages in the spleen, which are responsible for it being taken over byaged red blood cells. When hepcidin is insufficient due to mutations, iron deposits are formed. HH describes the genetic alterations of the hepcidin–ferroportin duo and not other conditions leading to metal storage [14].

However, the most severe forms of HH occur during childhood, being increasingly diagnosed in recent years [15], although in younger ages some other forms of genetic intolerances are associated with progressive liver disease [16].

Similarly to other increased plasma levels of metallic compounds, iron overload is toxic to the liver as it generates reactive oxygen species (ROS) and causes DNA damage which is further responsible for cell premature death [17]. Thus, the liver suffers a disbalance in its main functions, namely mitochondrial and lysosomal activity with a loss of antioxidant properties. Following hepatic cell apoptosis, an inflammatory response through specific cytokines and mediators transforms the cells into myofibroblasts that produce excess collagen, the mainstay in the future development of hepatic fibrosis, cirrhosis, and ultimately carcinoma [18]. However, studies have shown that a certain category of HH patients are more prone to severe liver disease progression, indicating the presence of independent risk factors. Thus, HH itself is a pro-inflammatory condition that participates in fibrogenesis through the activation of stellate cells by excess ferritin. The latter can predict the degree of fibrosis regardless of iron liver deposits and other known factors such as gender, previous steatosis or alcohol intake [19].

HH most typically affects males who are expected to develop a more prominent clinical picture, especially regarding the liver, due to associated comorbidities such as hepatitis C, fatty liver disease, or even alcohol consumption. Prevalence rates increase with age, and it appears that Northern Europe has an occurrence of up to 1 in 150 people. HH is less likely to affect non-Caucasian populations [20].

HH is inherited in an autosomal-recessive manner and the most frequently reported mutation is the homozygosity of C282Y on the HFE gene, being present in up to 80% of European patients with HH. The most recent classification of HH according to the molecular pattern was issued in 2022 by BIOIRON (International Society for the Study of Iron in Biology and Medicine) [4]. This working group transformed the previous four types of HH and divided them into the following subpopulations [4]:

1. HFE-related (C282Y homozygosity, compound heterozygosity or HFE deletion), in which secondary causes of excess iron should be investigated and tested for other genetic variants;
2. Non-HFE-related (hemojuvenil HJV-, hepcidin antimicrobial peptide HAMP-, transferrin receptor-2 TFR-2-, and ferroportin 1 SCL40A1-related) in which referral to specialized centers is advised, as is the commencement of phlebotomies;
3. Digenic (homozygosity and/or heterozygosity in two genes controlling iron homeostasis, either HFE or non-HFE), being difficult to interpret as pathogenic;
4. Molecularly undefined, in which the most common genes are not identified as mutated and specialized centers should take over.

According to the abovementioned classification, our patient belongs to the first subgroup, as the genetic test revealed C282Y/H63D compound heterozygosity.

Despite being largely used, genetic testing seems to spark controversy, especially in late-onset hemochromatosis or in disease forms where abnormal phenotypes are not identifiable. However, genetic testing remains of utmost relevance in neonatal disease. The Hemochromatosis and Iron Overload Screening (HEIRS) study included a significantly large cohort of patients in whom iron marker variability did not match genetic mutations, suggesting that disease phenotypes are not consistent [21]. On the other hand, genetic testing might lead to prompter diagnosis since screening tools are not feasible to use to detect a relatively rare disease.

HH with a confirmed HFE mutation exposes patients, especially males over 45 years of age, to much more severe liver disease progression, with fibrosis or cirrhosis being exhibited in up to a quarter of them. HFE-related HH comes with a four-fold higher risk of developing liver manifestations compared to the non-HFE HH [22]. The presented patient fits the described profile, with disease symptoms occurring at the age of 56 when liver involvement was found to be already significant. Moreover, homozygosity for C282Y exposes males to a greater-than-ten-fold risk of liver carcinoma. Since cirrhosis is a precedent of cancer development, this patient category should be monitored every six months via abdominal ultrasound, as detailed below [23].

However, rarer genetic variants exist and can predispose patients to iron overload despite lower penetration. C282Y/H63D compound heterozygosity was identified in 2–5% of North Europeans. This genotype was evaluated in a ten-year interval in 247 patients in North America, mostly males (63%) with a mean age of 50 years old. Briefly, 5.3% exhibited iron excess disease symptoms and four patients died during the follow-up because of HHC (3). Another recently published case emphasizes a display of HH symptoms in an 89-year-old patient with H63D heterozygosity. The patient presented with neurological symptoms (confusion, altered speech, and facial asymmetry), asterixis, and ascites, so cirrhosis was confirmed. MRI confirmed iron overload, and so did the serum markers. Cardiac ultrasound identified a dilation of the right atrial chamber. Phlebotomy was performed which markedly improved blood tests and the patient’s state [24]. Other rare forms of HH include the juvenile form (HJV or HAMP) which leads to iron accumulation in both boys and girls and disease symptoms in the first ten years of life.

Current recommendations indicate when to test for rarer gene variants in HH and that is in young patients with disease-evoking symptoms (heart or liver disease, or amenorrhea), patients with excess iron without a confirmation of cause, and in first-degree relatives of patients with confirmed HH with rare mutations [4]. Our patient did not meet the criteria for rare genetic testing, and he has no offspring; thus, no further gene examination was necessary in the family.

The main risk factors for HH-associated liver disease are summarized in Table 2.

In 2022, the European Association for the Study of Liver (EASL) released guidelines for HH, adding practitioners’ congruency in clinical practice [23]. Firstly, there is a clarification regarding patients who should be tested in a non-invasive manner for iron deposition. In cases of iron overload in serum levels, the recommendation highlights the importance of additional MRI in quantifying iron deposits in the liver but also in the brain, heart, and brain if the clinical setting is suggestive or in specific forms of hemochromatosis such as the juvenile HH. Alongside narrower availability and increased costs of this type of imaging, dedicated MRI software should be used. The level of iron overload has been suggested to be a predictor of organ damage but also of the treatment course via a prediction of the number of necessary phlebotomies.

Another point of interest in the EASL guidelines concerns the indications for a liver biopsy that have become very specific, namely to identify cirrhosis if other methods have previously failed, and to evaluate the degree of fibrosis if the ferritin level is over 1000 ug/L or there is an increase in transaminases. The staging of fibrosis is useful since it correlates with the iron level, but it is unlikely if ferritin levels are lower, and if no hepatomegaly, a low platelet count, or an increase in liver enzymes are detected. However, liver biopsy is not advised for the diagnosis of an iron overload condition [23].

An additionally proposed method to investigate liver fibrosis in HH is through transient elastography but larger cohorts are required to establish a better test threshold since it appears to be lower than that in other conditions such as infectious hepatitis or alcohol-induced or fatty liver damage [25]. Magnetic resonance elastography is not indicated in HH-related iron overload. Scores based on age and serum values of thrombocytes and transaminases such as FIB-4 can also be used to stage fibrosis in chronic liver disease but have not been validated in HH [26]. Despite these available techniques, there is no agreement upon the follow-up interval, threshold, or management plan based on large population studies.

**Figure 4:**
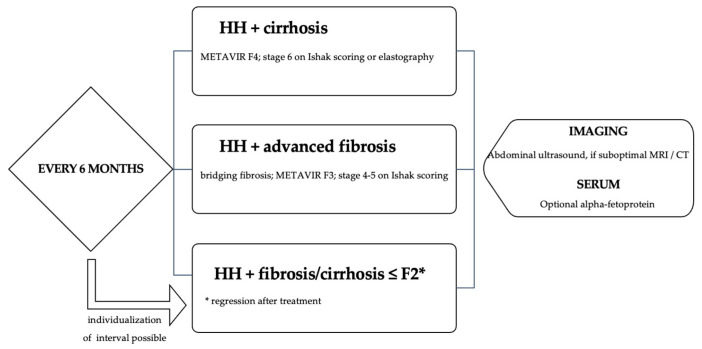
HHC screening and follow-up intervals in HH patients, adapted from [23].

The most undesirable risk in HH is developing end-stage liver disease and cancer, either hepatocellular carcinoma (HHC) or cholangiocarcinoma; thus, screening and regular monitoring are required. Current guidelines recommend the following (Figure 4) [23]:

A recent editorial published in Adams overviews current data on HHC related to HFE and refers to a study published by Natarajan et al. who aimed at identifying HHC risk factors according to the HFE genotype. The study was multicentric and included over 5000 patients with a mean follow-up period of 19 years which appears to be the longest available in the literature. Out of the 260 C282Y homozygotes, 11 developed HCC (4.2%) but most patients had ferritin levels below 1000 ug/L, which usually spares organ injury, and there were no results of liver elastography. In the study cohort, 8.1% of patients had cirrhosis prior to HHC development; however, four patients had no severe liver disease before cancer detection [7].

There have been other previous studies that evaluated the rate of HHC occurrence in HFE-C282Y male patients, with lengthy mean follow-up periods, from 8.9 years in the Atkins et al. analysis [27], to 17.7 years in Adams et al. from 2021 [28]. The rate of HHC detection varied from 0.9 to 3.2%.

The treatment of HH is currently standardized, despite the lack of scores for staging disease severity. Iron depletion is the standard of care and is conducted phlebotomies that can relocate iron and favor its use for erythrocyte formation [3]. The patient was treated accordingly, with regular phlebotomies with a median of 200 mL (150–300 mL) of blood per session and the adjustment of the frequency depending on the ferritin level. If treatment is initiated before the occurrence of cirrhosis of diabetes, mortality rates can be reduced. The procedure also helps in improving asthenia, joint pain, and liver enzymes. A proportion of patients benefit from phlebotomies in liver fibrosis regression but there is no evidence-based data on malignancy prevention or cardiac involvement. A second-line therapeutic option is erythrocyte apheresis but it remains available only in selected cases where phlebotomy has failed. Iron-chelating drugs (off-label deferasirox) can sometimes be used, usually in juvenile forms of the disease [29]. Deferasirox is a drug that enhances iron urine elimination by binding its trivalent form in the bloodstream. The newly formed ferrioxamine is not influenced by liver function since it is metabolized in the plasma. It is recommended to be administered via the intravenous or intramuscular route since bioavailability in the digestive tract is lower [30]. Therapy for HH requires an induction phase to rapidly reduce iron deposits followed by a maintenance period where treatment frequency can be adjusted according to ferritin levels, knowing that the target level is 50–100 ug/L [23].

The dilemma remains of whether or not iron depletion therapy via phlebotomy can prevent symptom onset, disease progression, or organ involvement since it is yet unclear whether or not iron excess itself is responsible for all the damage. A retrospective study published by Adams et al. in 2021 assessed HH-related comorbidities and mortality in C282Y-expressing patients undergoing phlebotomy. This population category showed a hazard increase of 44% in all-cause mortality, an eight-fold risk of experiencing HHC and a four-fold likelihood of cirrhosis [28]. Another recent study retrospectively analyzed 106 patients with C282Y mutations displaying stage 3 (F3) or 4 fibrosis (F4) regressed with treatment and exposed patients to a lower risk of primary liver malignancy [31].

HH represents a rare indication of liver transplantation (LT), with estimated rates at the publishing time of 1% out of 2266 LTs over 12 years at King’s College Hospital [32]. In the retrospective analysis, the diagnosis of HH was previously known or later confirmed via histopathology showing siderosis or genetic testing. The male/female ratio was 17/5, the median age was 55, and the participants underwent LT after cardiac evaluation. A significant number of patients were present/past alcohol consumers (11), and four had other liver conditions such as viral hepatitis or primary biliary cirrhosis. Apparently, only five had no associated harmful liver factors aside from HH. Survival rates at three months, and one and five years were 90%, 80.7%, and 74%, with no graft loss. Mortality in the study group was predominantly due to infectious complications. Neoplastic occurrence was found in four patients, with half showing metastatic HHC and the other two having prostate and pancreatic cancer [32].

The higher rate of mortality in HH due to infections might be explained by the influence of iron on the defense system, affecting cells responsible for the immune response, such as altering the CD4/CD8 ratio, causing low skin hypersensitivity, and causing an imbalanced response of the mononuclear cells in the peripheral blood to stimuli [32].

A number of publications report an increased rate of cancers in HH patients in other sites than the liver, namely pulmonary, pancreatic, or prostate [33]. However, incriminating iron overload in malignancies other than hepatic still requires consistent proof via larger cohort studies.

Regarding HH patients with end-stage liver failure, orthotopic liver transplants can be indicated in specialized centers for liver surgery [34]. Outcomes after liver transplantation in HH patients have been published in 2020 from a significant cohort (a total of 2590 patients out of which 29 had HH) [35]. Patients were followed-up for a ten-year period and underwent a liver biopsy to assess iron deposit rebuilding in the graft, and degree of the inflammation or fibrosis in the new organ. The mean age of HH patients was 52.7 years, there were 29 females versus 24 males, 27 had HH, and 2 had neonatal HH. Both serum iron values and ferritin levels decreased after the liver transplant. Briefly, 42% of patients showed iron excess upon the biopsy at three years, which later decreased. Stage 2 fibrosis was identified after liver transplant at the established time points (at 1, 3, 5, and 10 years) [35]. Despite the lack of proof of severe iron deposition in the organ graft, the survival rate of this patient category was significantly lower than that in the overall transplant population, with a mean survival rate of 45.5 months. The main reasons for death were severe infections and cardiovascular problems. The main issue is whether or not iron homeostasis is still responsible for HH patients’ poor survival even after successful orthotopic transplantation [35].

Acute liver failure is uncommon in HH. However, case reports have been described for congenital forms, iron supplementation in an undiagnosed case of HH, or overlapping sepsis, indicating the presence of a potential trigger in a fulminant setting [32].

The remaining issue is whether or not a HHC detection can be made earlier in HFE patients through imaging so that a surgical approach or organ transplantation could have a higher rate of success.

## 4. Conclusions

In conclusion, hemochromatosis is an iron storage disease that can be challenging to diagnose in the presence of unspecific symptoms and multiorgan involvement. This manuscript depicts our personal experience with a clinical case that presented with a “mix” of symptoms that we had to “match” to obtain the correct diagnosis, starting from the joints as rheumatologists. The importance of this case lies in the rarer genotype that the patient presented with and highlights atypical disease forms that should be in clinicians’ view. Nevertheless, the case brings into the spotlight the liver’s involvement, which may be present prior to the HH diagnosis and can be misleading as an etiology. Thus, we would like to emphasize the importance of a correct and prompt diagnosis leaning on current tools such as iron serum markers and imaging. Since liver injury is the most problematic organ involvement in HH and can worsen the prognosis via its progression to advanced fibrosis, cirrhosis, or hepatocellular carcinoma, special attention should be given to this organ’s involvement. A rapid initiation of phlebotomy sessions can halt the disease from further affecting the liver but also be beneficial for other symptoms such as fatigue, and arthralgia. Nevertheless, the multidisciplinary cooperation that HH patients need with rheumatologists, gastroenterologists, radiologists, hematologists, and genetic counseling is undeniable.

## Figures and Tables

**Table 1:** Patient’s blood tests dynamics regarding liver function and iron overload.

	Day 1	Day 5	Day 10 *	Day 20	Day 30
Hemoglobin (g/dL)	15.5	14.0	15.1	14.1	13.6
AST (U/L)	49	46	42	34	36
ALT (U/L)	24	38	33	27	37
Alkaline phosphatase (U/L)	153	127	132	110	95
Gamma glutamyl transferase	760	505	540	308	444
Bilirubin (md/dL)	1.98	2.1	1.76	1.1	1.7
INR	1.3	1.5	1.4	1.3	1.2
Platelets (10 × 3/μL)	146	116	110	130	141
Ferritin (ng/mL)		539	1000	875	280
Iron (μg/dL)		212			144
Transferrin saturation (%)		73.56			52
Total iron-binding capacity (μg/dL)		200			235

* on day 10 the first phlebotomy was performed.

**Table 2:** Risk factors for severe liver disease in HFE-related HH, adapted from [23].

Moderate to high alcohol consumption Diabetes Arthropathy Ferritin levels over 1000 ug/L Liver iron over 200 μmol/g Phlebotomy with more than 9.6 g of iron from stores

## Data Availability

Not applicable.
